# Postoperative Outcomes Are Comparable Between Arthroscopic Subscapularis Repairs Performed With Either All-Suture Anchors or Hard-Body Anchors

**DOI:** 10.1016/j.asmr.2024.101060

**Published:** 2024-12-05

**Authors:** Diego Gonzalez-Morgado, Javier Ardebol, Ali Ihsan Kilic, Matthew B. Noble, Lisa A. Galasso, Matthew Nugent, Cameron Phillips, Patrick J. Denard

**Affiliations:** a Oregon Shoulder Institute, Medford, Oregon, U.S.A.; b Orthopaedic Surgery Department, Hospital Universitari Vall d’Hebron, Universitat Autonoma de Barcelona, Barcelona, Spain; c Izmir Bakircay University, Izmir, Turkey

## Abstract

**Purpose:**

To compare clinical outcomes and tendon healing rates of patients undergoing arthroscopic rotator cuff repairs involving the subscapularis (SSC) tendon (isolated or combined) with all-suture anchors (ASAs) versus hard-body anchors (HBAs) and to compare SSC healing rates between these 2 anchors.

**Methods:**

A retrospective comparative study was performed on patients who underwent arthroscopic rotator cuff repair of the SSC with either ASAs or HBAs and had a minimum 2-year follow-up. Range of motion and patient-reported outcomes were collected pre- and postoperatively, including a visual analog scale for pain, American Shoulder and Elbow Surgeons score, and Subjective Shoulder Value. Postoperative strength was measured, including Constant strength and belly press test. SSC healing was evaluated on ultrasounds at the final follow-up.

**Results:**

Eighty-four patients met the study criteria. Twenty-eight underwent SSC repair with ASAs and 56 with HBAs. The mean follow-up for the ASA group and HBA group was 44 ± 22.7 months and 48.4 ± 28.3, respectively (*P* = .743). Baseline characteristics were comparable between groups (*P* > .05). Overall, patient-reported outcomes and range of motion showed significant improvements from baseline to the final follow-up in all groups (*P* < .001). Postoperatively, patients in the ASA group had greater improvement in forward flexion compared to the HBA group: 31° (95% confidence interval, 20°-42°) versus 14° (95% confidence interval, 5°-8°), respectively (*P* = .002). Postoperative Constant strength was higher in the ASA group compared to the HBA group: 17.5 ± 7.5 versus 13.5 ± 5.6, respectively (*P* = .04). No statistically significant difference in SSC retear rates was observed between groups: none in the ASA group and 3 (10.7%) in the HBA group (*P* = .27).

**Conclusions:**

Arthroscopic SSC repair leads to significant functional improvement, with both ASAs and HBAs demonstrating similar low failure rates.

**Level of Evidence:**

Level III, retrospective cohort study.

The subscapularis (SSC) is the strongest muscle of the rotator cuff. It is the primary internal rotator of the shoulder and contributes to dynamic stabilization of the glenohumeral joint.^1^^,^^2^ There is ongoing controversy regarding the optimal technique for repairing a torn SSC tendon. Recent advancements in implant design for shoulder arthroscopy have further fueled this debate, with the goal of finding the most effective fixation method.^3^

All-suture anchors (ASAs) have gained popularity for arthroscopic rotator cuff repairs (ARCRs) due to their advantages in bone preservation and easier revision compared to traditional hard-body anchors (HBAs).^4^ Studies have shown comparable biomechanical and clinical outcomes with ASAs when used for posterosuperior cuff repairs.^5^, ^6^, ^7^, ^8^, ^9^, ^10^, ^11^, ^12^, ^13^ However, research on SSC repairs specifically using ASAs remains limited.^8^^,^^10^^,^^11^^,^^14^, ^15^, ^16^ Considering the potential baseline differences in healing capacity between the SSC and the posterosuperior rotator cuff,^17^, ^18^, ^19^ as well as the lower bone density of the lesser tuberosity compared to the greater tuberosity,^20^ further investigation is needed to evaluate the potential advantages of ASAs in the repair of SSC tears.

This study aimed to compare the clinical outcomes of patients undergoing ARCR involving the SSC tendon (isolated or combined) with ASAs versus HBAs and to compare SSC healing rates between these 2 anchors. We hypothesized that SSC repair with ASAs would lead to similar functional outcomes but higher healing rates compared to HBAs.

## Methods

### Study Design

A retrospective review of prospectively collected data was carried out at a single institution between 2011 and 2021. Patients were included who underwent ARCR involving the SSC tendon (isolated or combined) with a minimum 2-year postoperative follow-up. Patients were excluded if there was a history of rotator cuff repair, proximal humerus fracture, or glenoid fracture. Groups were formed and compared based on the type of anchor used for the SSC repair: ASAs (FiberTak; Arthrex) versus HBAs (SwiveLock; Arthrex). Both anchor sutures consisted of an ultrahigh molecular weight polyethylene core surrounded by a braided polyester sheath with a silicone coating. Institutional review board approval was obtained before study commencement.

### Operative Technique and Arthroscopic Findings

All procedures were performed by a single high-volume, fellowship-trained shoulder surgeon (P.J.D.). Patients were positioned in the lateral decubitus position, and conventional portals were used. SSC tendon integrity was evaluated from the posterior viewing portal. SSC tear type was documented intraoperatively according to the Lafosse classification, and the cephalocaudal SSC tear size was reported as a percentage.^21^^,^^22^ Coracoplasty was performed if there was a narrowed subcoracoid space (<7 mm) or if a coracoid spur was present. From 2011 to 2018, all ARCRs were performed using HBAs, with a gradual shift to knotless suture anchors completed by 2017. Starting in 2018, there was a progressive transition to knotless ASAs. A single-row configuration was utilized for LaFosse type 1 and 2 tears. A double-row technique was employed for LaFosse type 3 and 4 tears, using ASAs or HBAs in the medial row and HBAs in the lateral row. In cases with posterosuperior cuff tears, limited acromioplasty was routinely conducted with preservation of the coracoacromial ligament. The mobility of the posterosuperior cuff was assessed, and an anterior interval slide was performed when necessary. Following release and adequate mobilization, the freed distal edges of the posterosuperior cuff were secured to the greater tuberosity using suture anchors in a double-row repair when possible. Alternatively, single-row or a single-row repair with a ripstop technique was done if it was determined that a double-row construct would create too much tension. All tears were fully repairable at the time of surgery.

Postoperatively, patients were immobilized in a sling for 4 to 6 weeks based on tear size to maintain neutral rotation and slight abduction. After sling discontinuation, patients were permitted to engage in passive external rotation (ER) and forward flexion (FF). Strengthening exercises and passive internal rotation (IR) were initiated at 8 to 12 weeks postoperatively, with a full return to activity, including sports activities, at 6 months.

### Study Variables

Demographic variables, including age, sex, body mass index, tobacco use, workers’ compensation status, traumatic or degenerative injury, and length of follow-up, were collected. Active range of motion (ROM) and patient-reported outcomes (PROs) were documented at baseline by the treating surgeon and postoperatively at a minimum 2-year follow-up by an independent surgeon blinded to the repair construct (A.I.K.). ROM measurements included FF, ER at the side, and IR. IR was numerically scaled based on the nearest spinal level achieved with the thumb (i.e., T10 = 10, T12 = 12, L2 = 14, L4 = 16, S1 = 18, hip = 20). PROs included the American Shoulder and Elbow Surgeons score, visual analog scale for pain score, and Subjective Shoulder Value. Isometric strengths were measured at a minimum 2-year follow-up using a manual muscle testing dynamometer with the patient in the standing position. To determine the Constant strength, the patient’s arm was positioned at a 90° FF, with their elbows fully extended and their forearms in a half pronation position. Constant strength was measured with the hand dynamometer placed on the dorsal aspect of the forearm, 2 cm proximal to the ulnar styloid process. The strength of the SSC muscle was evaluated using the belly press test.^23^ During the test, the patient was instructed to press the hand dynamometer between the palm and abdomen. For each participant, the average of the 3 independent measurements was taken. Return to previous activities, satisfaction, complications, and reintervention were included for analysis.

Magnetic resonance imaging for each patient was reviewed preoperatively by the operative surgeon (P.J.D.) and A.I.K. The degree of tendon fatty infiltration according to Goutallier was evaluated and recorded.^24^ The SSC was divided into the upper and lower half for grading based on the division described by Collin et al.^25^ The coracohumeral distance was evaluated by measuring the narrowest distance between the cortical border of the coracoid and the cortical border of the humeral head on T2-weighted axial images obtained from magnetic resonance imaging scans.^26^^,^^27^

Postoperatively, SSC tendon healing was graded using the Barth modification of the Sugaya classification^28^^,^^29^ for ultrasound assessment at the final follow-up by an orthopaedic surgeon (A.I.K.) trained in musculoskeletal ultrasound and blinded to the outcome. Grade I is defined by tendons with sufficient thickness (>2 mm) and normal echo-structure. Grade II indicates sufficient thickness with partial hypo-echogenicity. Grade III tendons have insufficient thickness (<2 mm) without discontinuity. Grade IV repairs show minor discontinuity in the tendon. Grade V repairs show major discontinuity. Grade I, II, and III repairs were considered healed. Grade IV and V repairs were considered retears.

### Statistical Analysis

Each patient with SSC repair using ASAs was matched to 2 patients whose SSC was repaired using HBAs. For equal comparison, matches were made based on age (a range of 5 years), sex, rotator cuff characteristics, preoperative FF (range of 10°), ER (range of 5°), IR (range of 1 vertebral level), American Shoulder and Elbow Surgeons score (range of 10), Subjective Shoulder Value (range of 10), and visual analog scale for pain (range of 2). Continuous data were reported as a mean with standard deviation with comparison by the Student *t* test or Mann-Whitney *U* test according to normality. Categorical data were reported as frequencies and percentages with comparison by χ^2^. All statistical analyses were conducted using SPSS version 25 (SPSS, Inc.). A *P* value threshold of .05 was used to denote statistical significance.

## Results

A total of 84 patients met the study criteria: 28 had an SSC repair with ASA anchors and 56 with HBA. The mean follow-up for the ASA group and HBA group was 44 ± 22.7 months and 48.4 ± 28.3 months, respectively (*P* = .743). Nine patients (32.1%) in the ASA group and 15 (26.8%) in the HBA group had an isolated SSC tear. Baseline characteristics are summarized in Table 1.

**Table 1:** Baseline Characteristics of Arthroscopic Rotator Cuff Repair With Subscapularis Tendon Involvement

Characteristic	ASA (n = 28)	HBA (n = 56)	*P* Value
Patient demographics			
Age, y	56.3 ± 11.6	58.4 ± 8.5	.401
Sex: male	16 (57.1)	36 (64.3)	.525
BMI	29.2 ± 7.5	29.6 ± 5.1	.801
Tobacco use: yes	3 (10.7)	10 (17.9)	.529
Workers’ compensation: yes	7 (25)	12 (21.4)	.712
Traumatic injury: yes	18 (64.3)	31 (55.4)	.434
Follow-up: months	44 ± 22.7	48.4 ± 28.3	.743
MRI findings			
Goutallier classification: grade 0-1-2-3-4 (n)			
SS fatty infiltration	16-5-5-1-1	34-8-12-2-0	.667
IS fatty infiltration	20-6-2-0-0	33-10-12-1-0	.351
Upper SSC fatty infiltration	15-10-3-0-0	30-17-9-0-0	.252
Lower SSC fatty infiltration	26-2-0-0-0	47-5-4-0-0	.334
CHD: mm	6.7 ± 2.2	6.9 ± 1.6	.714
Intraoperative findings			
Isolated SSC tear: yes	9 (32.1)	15 (26.8)	.608
Lafosse classification: grade I-II-III-IV-V (n)	15-10-3-0-0	29-19-8-0-0	.9
Cephalocaudal SSC tear size: %	41.1 ± 18.1	39.1 ± 15.8	.61
Repair			
SSC repair technique:			.863
Single-row: yes	20 (71.4)	41 (73.2)	
Double-row: yes	8 (28.6)	15 (26.8)	
Total number of anchors: mode (range)	2 (1-6)	2 (1-4)	
Coracoplasty: yes	7 (25)	18 (32.1)	.5

NOTE. Values are presented as mean ± SD or n (%) unless otherwise indicated. Bold values indicate statistical significance (*P* < .05).

ASA, all-suture anchors; BMI, body mass index; CHD, coracohumeral distance; HBA, hard-body anchors; IS, infraspinatus; MRI, magnetic resonance imaging; SS, supraspinatus; SSC, subscapularis.

Preoperative and postoperative outcomes are summarized in Table 2. Overall, PROs and ROM showed significant improvements from baseline to the final follow-up in all groups (*P* < .001). Patients from the ASA group experienced greater improvement in FF from preoperative to the last follow-up compared to the HBA group: 31° (95% confidence interval, 20°-42°) versus 14° (95% confidence interval, 5°-8°), respectively (*P* = .002), but final FF was comparable between groups: 158° ± 15 for the ASA group and 154° ± 10 for the HBA group (*P* = .565). Postoperative Constant strength was higher in the ASA group compared to the HBA group: 17.5 ± 7.5 versus 13.5 ± 5.6, respectively (*P* = .04). Eighteen and 28 patients from the ASA and HBA groups, respectively, underwent ultrasound healing evaluation. No SSC retears were found in the ASA group, whereas 3 retears (10.7%) were observed in the HBA group. Three complications were noted, comprising failure of posterosuperior cuff healing with anchor pullout: 1 in the ASA group and 2 in the HBA, all resulting in conversion to reverse shoulder arthroplasty. Postoperative satisfaction was achieved in 23 patients (82.1%) from the ASA group and in 53 patients (94.6%) from the HBA, with no significant difference between groups (*P* = .385).

**Table 2:** Comparison of Functional Outcomes of Subscapularis Tendon Repair With All-Suture Anchors and Hard-Body Anchors

Characteristic	ASA (n = 28)	HBA (n = 56)	*P* Value
VAS			
Preoperative (mean ± SD)	5.6 ± 2.1	5.5 ± 2	.846
Postoperative (mean ± SD)	1.9 ± 2.4	1.3 ± 1.8	.269
Improvement (mean, 95% CI)	3.7 (2.8-4.5)	4 (3.5-4.8)	.386
*P* value	**<.001**	**<.001**	
ASES			
Preoperative (mean ± SD)	42.3 ± 13.6	43.3 ± 15.2	.768
Postoperative (mean ± SD)	84.1 ± 18.1	88.5 ± 12.7	.258
Improvement (mean, 95% CI)	41.8 (36.9-46.7)	45.2 (40.6-49.9)	.363
*P* value	**<.001**	**<.001**	
SSV			
Preoperative (mean ± SD)	35.6 ± 22.9	38 ± 18.2	.606
Postoperative (mean ± SD)	86.4 ± 14.9	88.8 ± 14.8	.503
Improvement (mean, 95% CI)	50.1 (42.4-59.4)	50.8 (44.5-57.1)	.989
*P* value	<.001	<.001	
Active FF			
Preoperative (mean ± SD)	128° ± 28°	139° ± 25°	.104
Postoperative (mean ± SD)	158° ± 15°	154° ± 10°	.565
Improvement (mean, 95% CI)	31° (20-42)	14° (5-18)	**.002**
*P* value	**<.001**	**<.001**	
Active ER			
Preoperative (mean ± SD)	53° ± 13°	55° ± 12°	.391
Postoperative (mean ± SD)	68° ± 8°	67° ± 10°	.902
Improvement (mean, 95% CI)	15° (9-21)	12° (4-12)	.68
*P* value	**<.001**	**<.001**	
Active IR∗			
Preoperative (mean ± SD)	L5 ± 2	L4 ± 2	.063
Postoperative (mean ± SD)	T11 ± 3	T12 ± 3	.507
Improvement (mean, 95% CI)	5 (3-6)	3 (3-4)	.078
*P* value	**<.001**	**<.001**	
Postoperative SSC strength: lbs. (mean ± SD)	23.1 ± 7.8	20.7 ± 8	.328
Postoperative Constant strength: lbs. (mean ± SD)	17.5 ± 7.5	13.5 ± 5.6	**.04**
US evaluation of SSC integrity	n = 18	n = 28	.162
Sugaya classification			
Type I, n (%)	12 (66.7)	10 (35.7)	**.04**
Type II, n (%)	5 (27.8)	12 (42.9)	.301
Type III, n (%)	1 (5.5)	3 (10.7)	.544
Type IV, n (%)	0	3 (10.7)	.27
Type V, n (%)	0	0	
Retear (Sugaya IV and V)	0	3 (10.7)	.27
Return to activity, n (%)	23 (82.1)	49 (87.5)	.465
Satisfaction, n (%)	25 (89.3)	53 (94.6)	.385
Complication, n (%)	1 (3.6)	2 (3.6)	1
Reintervention, n (%)	1 (3.6)	2 (3.6)	1

NOTE. Bold values indicate statistical significance (*P* < .05).

ASA, all-suture anchors; ASES, American Shoulder and Elbow Surgeons; ER, external rotation at side; FF, forward flexion; HBA, hard-body anchors; IR, internal rotation; SSC, subscapularis; SSV, Subjective Shoulder Value; US, ultrasound; VAS, visual analog scale.

∗ Spinal level.

## Discussion

The principal finding of this study was that arthroscopic SSC repair of isolated SSC tears and combined rotator cuff tears led to significant improvement in PROs and ROM at a minimum 2-year follow-up, regardless of whether ASAs or HBAs were used. Improvement in FF and postoperative Constant strength were higher in the ASA group. Patient satisfaction and return to previous activity were observed in most patients without significant differences between groups.

ASAs have become popular in ARCR because of their small size, bone stock preservation, and ease of revision.^4^ Biomechanical and clinical studies comparing ASAs with HBAs for ARCR have demonstrated similar outcomes between both anchors.^5^, ^6^, ^7^, ^8^, ^9^, ^10^, ^11^, ^12^, ^13^ Cadaveric studies by Ntalos et al.^9^ and Bernardoni et al.^5^ found no difference in load to failure between ASAs and HBAs in single-row and double-row fixation, respectively. Despite the rising number of clinical studies on ASA for ARCR, none have focused on SSC tears. Di Gennaro et al.^6^ compared clinical outcomes of ASAs and HBAs for supraspinatus repairs in 60 patients and found noninferior performance in Constant scores at the last follow-up (ASAs: 89.33 vs HBAs: 83.27, Δ5.2, *P* = .057). Similarly, Yan et al.^12^ conducted a randomized control trial comparing the Constant-Murley score between ASAs and HBAs for ARCR, but the authors did not specify whether SSC tears were included. They found no significant differences in Constant-Murley scores between the 2 groups (ASAs: 86.2 vs HBA: 86; *P* = .122). Although research on the clinical application of ASAs continues to grow,^7^^,^^8^ a critical gap exists in our understanding of their effectiveness for SSC tears. SSC repairs may pose additional challenges, partly due to the lower bone density of the lesser tuberosity compared to the greater tuberosity, which may increase the risk of fixation failure with ASAs.^20^ In addition, the healing potential of the SSC may differ from that of the posterosuperior rotator cuff. Bartl et al.,^17^ for instance, reported a retear rate of 4% for the SSC compared to a 19% retear rate for the supraspinatus in anterosuperior rotator cuff repairs. Furthermore, SSC retears are often observed following posterosuperior cuff retears, as noted by Lee et al.^19^ These findings highlight the need for further research to evaluate SSC repair outcomes when using ASAs. Our study directly addressed this need by investigating the clinical utility of ASAs in repairing SSC tears. It is worth noting that groups were matched, allowing us to homogenize baseline characteristics between groups, and this increased the attributability of the results to the type of anchor used.

SSC repair significantly improves ARCR outcomes by restoring shoulder stability, function, and the successful healing of other cuff tendons.^1^, ^2^, ^3^ Based on prior clinical studies demonstrating comparable outcomes between ASAs and HBAs in ARCR,^6^^,^^11^^,^^12^^,^^16^ we anticipated no significant differences in clinical outcomes for our study. Consistent with previous studies, PROs significantly improved following surgery in both groups. However, our study found that FF improvement and postoperative Constant strength were significantly higher in the ASA group compared to the HBA group. Additionally, both IR improvement and IR strength were higher in the ASA group compared to the HBA group, although statistical significance was not achieved. This could be attributed to the limited sample size. One possible explanation for the slight differences in ROM and postoperative strength favoring the ASA group could be the higher healing rate in the ASA group, although this is difficult to state conclusively with the small numbers in each group and lack of statistical difference in healing rates.

Clinical studies utilizing ASAs for ARCR report variable retear rates, ranging from 2% to 30%.^8^^,^^10^, ^11^, ^12^^,^^14^, ^15^, ^16^ Yan et al.^12^ found a similar retear rate between ASAs (5.7%) and HBAs (1.9%), *P* = .618. Piatti et al.^10^ compared ASAs versus metallic anchors in small and medium supraspinatus tears, finding a 95% rate of Sugaya I and II healing status in the ASA group compared to 85% in the metallic anchor group. Similarly, Ro et al.^14^ studied ASA fixation in small and medium posterosuperior cuff tears using a single-row repair technique. The authors reported a 67.7% healing rate (Sugaya I and II) with only 2 retears. Although their Sugaya I and II rates were lower than our 94.5% rate, direct comparison is limited due to the differing tendons studied. Our study found a higher rate of complete healing with ASAs compared to the HBAs, with only 3 retears observed in our HBA group, which aligns with previously reported healing rates ranging from 83% to 100%.^30^^,^^31^ Despite the limited study size and lack of statistical significance, these findings could suggest that ASAs may facilitate tendon healing. Most ASAs are 50% to 40% the size of HBAs in diameter. The reduction in footprint violation with ASAs may be a biologic advantage in that they allow greater contact area between the repaired tendon and bone per point of fixation.^4^ This translates to enhanced tendon-to-bone contact, promoting improved healing and potentially contributing to the restoration of both motion and strength.^4^^,^^32^^,^^33^ While theoretical, at minimum, it appears that ASAs have no disadvantage compared to HBAs in SSC repairs.

Rotator cuff retear rates vary from 13% to 94%, and while not all cause symptoms, some patients may need revision surgery.^34^, ^35^, ^36^ While evidence on the functional benefits of ASAs for primary repair is limited, they also offer advantages for potential revision surgery. ASAs may simplify rotator cuff revision surgery. During a revision, HBAs can be inserted in ASAs’ location without the need for anchor removal. It has also been demonstrated that if pullout occurs, ASAs cause less disruption to the bone and can be salvaged using HBAs without compromising the biomechanical performance.^9^^,^^37^ Notably, in the current study, anchor pullout with ASAs was rare, occurring in only 1 case, whereas it occurred in 2 cases with HBAs.

### Limitations

This study has several limitations. First, the study’s retrospective nature may increase the risk of selection and information biases. Second, while there was gradual adoption by the operative surgeon for ASAs and these anchors are currently utilized regardless of bone quality in his practice, the anchor type was ultimately selected at the surgeon’s discretion, which could have introduced bias. Additionally, since ASAs were implemented later in the study period, improved outcomes could have been influenced by the surgeon’s increased expertise or measurement changes over time. Third, the generalizability of our findings may be limited since all surgeries were performed by a single high-volume shoulder surgeon at a single institution. Fourth, the preoperative and postoperative imaging assessment conducted by a single surgeon, without multiple graders and an inter-rater correlation study, is susceptible to biases. Fifth, this study focused on rotator cuff tears with SSC involvement, ranging from isolated SSC tears to combined rotator cuff tears, but we did not investigate the impact of the different anchors on supraspinatus and infraspinatus tendons. This might make it challenging to generalize our results to all rotator cuff tear patterns and combinations. Finally, given the short-term nature of this study’s follow-up, predicting the mid- and long-term comparative results remains uncertain.

## Conclusions

Arthroscopic SSC repair leads to significant functional improvement, with both ASAs and HBAs demonstrating similar low failure rates.

## Disclosures

The authors declare the following financial interests/personal relationships which may be considered as potential competing interests: P.J.D. is a consultant or advisor for Arthrex, receives speaking and lecture fees from 10.13039/100007307Arthrex, and receives royalties from Arthrex. All other authors (D.G-M., J.A., A.I.K., M.B.N., L.A.G., M.N., C.P.) declare that they have no known competing financial interests or personal relationships that could have appeared to influence the work reported in this paper.
